# Cerium-Promoted Ginsenosides Accumulation by Regulating Endogenous Methyl Jasmonate Biosynthesis in Hairy Roots of *Panax ginseng*

**DOI:** 10.3390/molecules26185623

**Published:** 2021-09-16

**Authors:** Ru Zhang, Shiquan Tan, Bianling Zhang, Pengcheng Hu, Ling Li

**Affiliations:** 1 Hunan Institute of Engineering, College of Materials and Chemical Engineering, Xiangtan 411104, China; tanshiquan11@163.com (S.T.); blzhang369@163.com (B.Z.); hpc2211899409@163.com (P.H.); liling20180119@126.com (L.L.); 2 Hunan Provincial Key Laboratory of Environmental Catalysis and Waste Recycling, Hunan Institute of Engineering, Xiangtan 411104, China; 3 Hunan International Joint Laboratory of Animal Intestinal Ecology and Health, College of Life Sciences, Hunan Normal University, Changsha 410081, China

**Keywords:** cerium, oxidative stress, reactive oxygen species, MeJA, *Panax ginseng*, ginsenosides

## Abstract

Among rare earth elements, cerium has the unique ability of regulating the growth of plant cells and the biosynthesis of metabolites at different stages of plant development. The signal pathways of Ce^3+^-mediated ginsenosides biosynthesis in ginseng hairy roots were investigated. At a low concentration, Ce^3+^ improved the elongation and biomass of hairy roots. The Ce^3+^-induced accumulation of ginsenosides showed a high correlation with the reactive oxygen species (ROS), as well as the biosynthesis of endogenous methyl jasmonate (MeJA) and ginsenoside key enzyme genes (*PgSS*, *PgSE* and *PgDDS*). At a Ce^3+^ concentration of 20 mg L^−1^, the total ginsenoside content was 1.7-fold, and the total ginsenosides yield was 2.7-fold that of the control. Malondialdehyde (MDA) content and the ROS production rate were significantly higher than those of the control. The activity of superoxide dismutase (SOD) was significantly activated within the Ce^3+^ concentration range of 10 to 30 mg L^−1^. The activity of catalase (CAT) and peroxidase (POD) strengthened with the increasing concentration of Ce^3+^ in the range of 20–40 mg L^−1^. The Ce^3+^ exposure induced transient production of superoxide anion (O_2_^•^^−^) and hydrogen peroxide (H_2_O_2_). Together with the increase in the intracellular MeJA level and enzyme activity for lipoxygenase (LOX), there was an increase in the gene expression level of MeJA biosynthesis including *PgLOX*, *PgAOS* and *PgJMT*. Our results also revealed that Ce^3+^ did not directly influence *PgSS*, *PgSE* and *PgDDS* activity. We speculated that Ce^3+^-induced ROS production could enhance the accumulation of ginsenosides in ginseng hairy roots via the direct stimulation of enzyme genes for MeJA biosynthesis. This study demonstrates a potential approach for understanding and improving ginsenoside biosynthesis that is regulated by Ce^3+^-mediated signal transduction.

## 1. Introduction

For thousands of years, *Panax ginseng* has been one of the most valued herbal medicines in oriental countries [1]. The major active ingredients of ginseng are ginsenosides, polysaccharides, peptides and phenolic compounds, which have been proven to possess important clinical effects [2,3,4,5]. Because the cultivation period of ginseng is long and the active ingredients mainly accumulate in aged roots, it is sensible to efficiently culture ginseng cells or tissues in high yield [6,7]. However, ginsenoside accumulation is the result of ginseng’s long-term interaction with the environment, and the cultivation of ginseng is susceptible to environmental stresses, nutrients, etc., during the growth of ginseng.

Rare earth elements (REEs) denote a group of 17 metallic elements with similar chemical properties. It has been reported that REEs have important regulatory effects on plant physiology [8,9]. An appropriate amount of REEs not only promotes the photosynthesis rate, root development and increase in biomass, but also improves plant resistance against stress by altering the activities of some antioxidant enzymes, such as superoxide dismutase (SOD), catalase (CAT) and peroxidase (POD) [10,11]. For example, trivalent lanthanum (La^3+^) protects soybean plants from oxidative stress by regulating reactive oxygen species (ROS) or improving their defense ability mediated by the antioxidant system [12]. La^3+^ also alleviates the oxidative damage induced by UV-B radiation through the reduction of H_2_O_2_ and O_2_^•^^−^ content [12,13]. Furthermore, trivalent cerium (Ce^3+^) in an appropriate concentration could enhance the defense system as well as increase the length, number and volume of the root, leading to higher fresh and dry weights of the root and shoot [8,14]. Ce^3+^ can also stimulate flavonoid biosynthesis by inducing PAL activity, which triggers oxidative defense responses in *Tetrastigma hemsleyanum* suspension cells [15]. It was reported that La^3+^ and Ce^3+^ can accelerate the regeneration of *Anoectochilus roxburghii* cultured in vitro [16]. Furthermore, the rooting rate and root length of peach plantlets could be increased through growth in a Ce^3+^-supplied medium [17]. All the results show that at optimized conditions, Ce^3+^ can promote plant growth and physiological properties in tissue or callus culture.

Under environmental stimuli, there is a generation of ROS, including hydrogen peroxide (H_2_O_2_), superoxide anion (O_2_^•^^−^), singlet oxygen (^•^O_2_) and hydroxyl radical (OH^•^), in plant cells [18,19]. Moreover, ROS can act as a signal molecule in plants and trigger a series of cellular responses. Some enzymatic antioxidants in plants can scavenge ROS for environment adaption [20]. Antioxidant enzymes in ginseng mainly include SOD, CAT, POD and ascorbate peroxidase (APX), which can regulate ROS levels in fluctuating environments [18,21]. It was reported that ROS and antioxidant enzymes related to oxidative stress could be coupled with the accumulation of ginsenosides in OGA-induced ginseng cells or MeJA-treated ginseng adventitious roots [22,23,24]. Linoleic acid, a precursor of jasmonates (JAs) biosynthesis that stimulates the activities of SOD, CAT and APX in ginseng adventitious roots, could act as an indicator of ROS generation under elicitation [25]. Moreover, elevated activities of SOD, POD and APX associated with increased ginsenosides production were found in adventitious roots after elicitation with nitroprusside, a nitric oxide donor [26]. It is probably that ROS may function as signal molecules for the induction of defense genes and thus can stimulate the production of ginsenosides as defense metabolites [27]. However, the related mechanism is still unclear.

It is well known that signal molecules such as JAs can act as pivotal elicitors to induce secondary plant metabolites. In plant culture, using signal components as elicitors has become an effective strategy to produce target secondary metabolites. It has been demonstrated that ROS could act as an oxidative stress molecule to trigger the biosynthesis of JAs [27]. These suggest that if we find a chemical that can appropriately activate ROS production or directly promote endogenous JAs biosynthesis, it may become a promising molecule to enhance the production of secondary plant metabolites. As far as we know, no previous investigation has been performed regarding the effect of cerium application on ginseng tissue or cell culture and the evaluation of phytochemicals in vitro. The mechanisms of secondary metabolites under this condition are not clear. Therefore, this study attempts to investigate the effects of different concentrations of cerium on ginseng growth, environmental adaptability and active ingredients’ accumulation in terms of antioxidant defense or ROS regulation.

## 2. Results and Discussion

### 2.1. Ginseng Hairy Roots Formation

**Figure 1:**
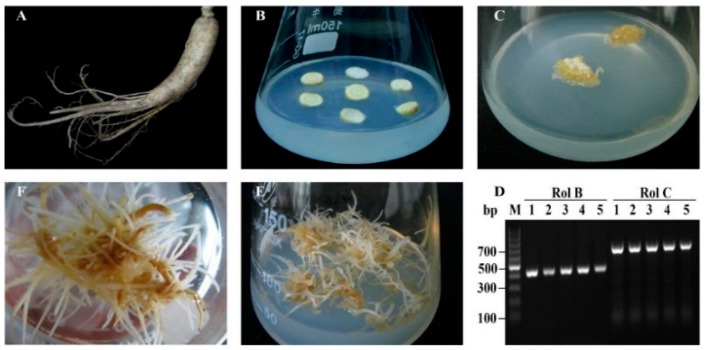
Hairy root induction from *P. ginseng.* (**A**) Fresh 4-year root of ginseng. (**B**) Explants from the fresh 4-year root. (**C**) Putative hairy roots appeared after 4 weeks of culture induced by *A. rhizogenes* A4. (**D**) Hairy root lines with normal phenotypic characteristics were confirmed by RT-PCR using the landmark genes rolB and rolC. Screened hairy roots were cultured on solid (**E**) and liquid (**F**) ½ MS mediums.

The cultivation of hairy roots is an effective method to obtain biologically active compounds and to investigate gene functionalities, especially for slow-growing ginseng plants. As an attractive approach for fast growth, fresh root segments of ginseng maintained in vitro were infected with *Agrobacterium rhizogenes* A4 (Figure 1A,B). The putative hairy roots appeared after 4 weeks (Figure 1C) and were excised from the explants and cultured in a ½ MS medium containing cefotaxime at 2-week subculture intervals for further selection. As confirmed by RT-PCR (Figure 1D), the hairy root lines presented normal phenotypic characteristics. All five lines (Figure 1D, lane 1–5) harbored the transcripts of landmark *rolB* and *rolC* genes. The results suggest that the ginseng hairy roots were successfully transformed by *A. rhizogenes* A4. In ½ MS medium, most of the transformed hairy roots showed vigorous elongation with several lateral roots. Among the five hairy root lines, the line that showed the fastest growth and yielded more lateral roots was cultured on solid (Figure 1E) as well as liquid (Figure 1F) ½ MS mediums for further studies.

### 2.2. Growth Index of Ginseng Hairy Roots

**Figure 2:**
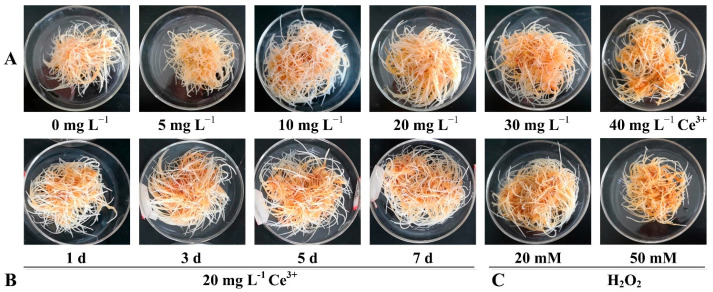
Growth and morphology of ginseng hairy roots under different treatments. (**A**) After a preculture period of 21 d, followed by Ce^3+^ treatment of different concentrations for 7 d in a liquid ½ MS medium. (**B**) After a preculture period of 21 d, followed by Ce^3+^ treatment of 20 mg L^−1^ for 1, 3, 5 and 7 d in a solid ½ MS medium. (**C**) After a preculture period of 21 d, followed by H_2_O_2_ treatment at different concentrations for 7 d in a liquid ½ MS medium.

The effect of Ce^3+^ concentration on morphogenic changes, root elongation, biomass and the growth ratio of ginseng hairy roots are presented in Figure 2 and Table 1. At Ce^3+^ concentrations of 5, 10 and 20 mg L^−1^, the ginseng hairy roots were light yellow, long and branched, whereas at 30 and 40 mg L^−1^, they were slightly darkened in color, short and fragile. Taking a concentration of Ce^3+^ of zero as the control, root elongation was the highest at 10 mg L^−1^ (1.4-fold of control) and the lowest at 40 mg L^−1^ (0.9-fold of control). To determine the biomass, dry matter content (DMC) and growth ratio (GR), as well as the fresh weight (FW) and dry weight (DW), the hairy roots sampled on the seventh day of Ce^3+^ treatment were assayed. At a Ce^3+^ concentration of 10 mg L^−1^, FW and DW were 16.0 g and 1.7 g, respectively, while GR was 13.9, the highest as depicted in Table 1. When the Ce^3+^ concentration was 40 mg L^−1^, the FW, DW and GR values became the smallest, reduced by 12.0%, 12.5% and 16.3% compared to the control. It was noted that the DMC value was the highest at a Ce^3+^ concentration of 20 mg L^−1^. The GR was the lowest at a Ce^3+^ concentration of 40 mg L^−1^, suggesting high matter loss at a high Ce^3+^ dosage. As a comparison, treatment with 20 mM H_2_O_2_ did not affect the growth of hairy roots, but that with 50 mM H_2_O_2_ not only caused color darkening but also induced negative effects on the elongation of hairy roots and accumulation of biomass.

REEs have been widely applied in plant biotechnology, and an appropriate amount of REEs could have positive effects on callus growth, cell viability, seed germination and root development [15,16,28,29]. Our results so far indicate that Ce^3+^ can significantly affect the metabolic processes of ginseng hairy roots. Treatment of Ce^3+^ in low concentrations (5–20 mg L^−1^) improved the growth of ginseng hairy roots, whereas that in high concentrations (above 30 mg L^−1^) inhibited the growth, partially consistent with reported literature [8,14,30]. It was reported that cerium has certain effects on plant growth or physiology. A high concentration of Ce^4+^ nanoparticles (2000 mg L^−1^) did not affect radish root elongation [31], whereas Ce^4+^ nanoparticles at 500 mg L^−1^ could increase the elongation of the cucumber root and alfalfa stem [32]. Cerium treatment in magnesium-deficient media significantly promoted the activities of key enzymes as well as the contents of amino acids, chlorophyll, soluble proteins and spinach growth [33]. The reports suggest that the promotional effect of cerium could be related to the state of cerium as well as the specificity of plant species and growth environment.

### 2.3. Ce^3+^ Stimulates Ginsenoside Accumulation in Ginseng Hairy Roots

At present, more than 50 ginsenosides have been isolated and identified from ginseng roots. The major ones are Rb_1_, Rb_2_, Rc, Rd, Re and Rg_1_, constituting more than 80% of total ginsenosides. These six ginsenosides can be used as a representative for the analysis of total saponins [34]. As shown in Table 2, the ginsenoside content increased significantly with the increase in Ce^3+^ concentration from 5 to 20 mg L^−1^, after which ginsenoside content started to decrease. Under the Ce^3+^ treatment of 20 mg L^−1^, the content of ginsenoside Rb_1_, Rb_2_, Rc, Rd, Re and Rg_1_ increased by 1.7-, 3.4-, 3.3-, 2.1-, 1.4- and 1.2-fold, respectively, compared with the control of a Ce^3+^ concentration of zero. The total ginsenoside reached 16.4 mg g^−1^, which is a 1.7-fold increase, giving a total yield 2.7-fold greater than that of the control. Despite the fact that the total ginsenoside content did not change much upon Ce^3+^ treatment of 30 mg L^−1^, there was a significant decrease in total yield. It is plausible that Ce^3+^ inhibited the growth of ginseng hairy roots, resulting in an increase in the relative content of ginsenosides in hairy roots. When the concentration of Ce^3+^ was 40 mg L^−1^, the production of ginsenoside decreased drastically, by 7.9% compared with the control value.

In fact, studies have revealed that REEs showed various effects on the production of secondary metabolites in plant cultures. A Ce^3+^ supplement on the solid culture medium of *Saussurea medusa* cells not only improved the biomass, but also increased the total flavonoids, and its highest biomass and yield increased by 70% and 100% compared to those of the control, respectively [35]. Cerium was found to induce apoptosis and was used as an effective abiotic elicitor to bring about a 5-fold taxol increase in comparison to that of the control [35,36,37]. A similar study indicated that at low concentrations (0.1 mM), cerium did not affect taxol biosynthesis, but at high concentrations (1 mM), cerium induced apoptosis and taxol biosynthesis in *Taxus cuspidate* suspension cultures [38]. The addition of cerium (CeO_2_ or CeCl_3_) to the suspension cells of *Catharanthus roseus* increased the content of indole alkaloids, ajmalicine or catharanthine [39]. Furthermore, it was reported that low-concentration lanthanum directly induced the key enzyme genes of tanshinone, which resulted in enhanced rosmarinic acid and salvianolic acid B 129% and 148% more than the control in the hairy roots of *Salvia miltiorrhiza*, respectively [40]. Because of the significant promotion effect of cerium on the accumulation of ginsenoside in ginseng hairy roots, it becomes imperative to understand the secondary metabolic response induced by cerium. Furthermore, the results suggest that Ce^3+^ increases the content of ginsenosides by regulating the biosynthetic metabolic flow of ginsenosides.

### 2.4. Ce^3+^-Induced ROS Production and Antioxidant Enzyme Activities

**Figure 3:**
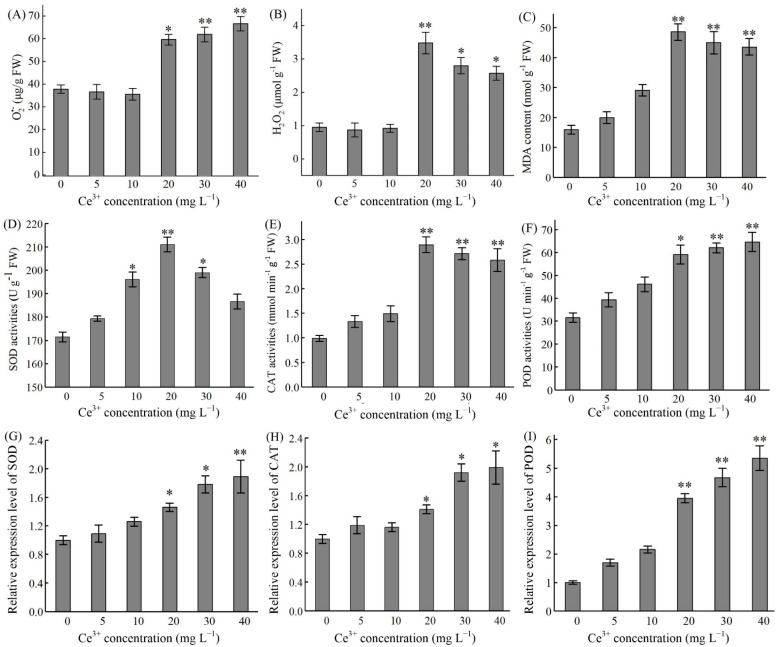
ROS accumulation and antioxidant enzymes activities of ginseng hairy roots upon Ce^3+^ treatment of 7 d in a ½ MS medium after a 21 d preculture. (**A**–**C**) Content of O_2_^•^^−^, H_2_O_2_ and MDA. (**D**–**F**) Antioxidant enzymes activities of SOD, CAT and POD. (**G**–**I**) Relative expression level of *SOD*, *CAT* and *POD* genes. The differences between the treated hairy roots and control hairy roots (0 h) are statistically significant (* *p* < 0.05, ** *p* < 0.01).

ROS accumulation is a hallmark of stress in plants, and O_2_^•^^−^, H_2_O_2_ and malondialdehyde (MDA) are frequently used as an index of oxidative stress. As shown in Figure 3A, Ce^3+^ addition (20–40 mg L^−1^) resulted in a significant increase in O_2_^•^^−^ content in ginseng hairy roots after 7 d treatment (*p* < 0.05) and was 1.8-fold higher at 40 mg L^−1^ than that of the control. Furthermore, Ce^3+^ (20–40 mg L^−1^) rapidly elicited H_2_O_2_ synthesis and released H_2_O_2_ in ginseng hairy roots, which peaked at a Ce^3+^ concentration of 20 mg L^−1^ (Figure 3B). The response of MDA content to Ce^3+^ was like that of H_2_O_2_. At a concentration of 20 mg L^−1^, Ce^3+^ contributed to a maximal increase in MDA, which was 3.1-fold higher than that of the control (Figure 3C).

The generation of ROS is a common event in plant stress response, resulting in lipid peroxidation, which could damage the membrane and cause changes to plant growth as well as to metabolic and physiological processes [41,42]. It was found that the positive or negative effects of REEs on the physiological metabolism of plant cells mainly depend on REEs dosage such as in the case of Ce^3+^ [43,44]. High dosages of REEs may cause the generation of ROS and lead to oxidative stress in plant cells [45]. It was reported that La^3+^ protected soybeans from oxidative stress by reacting with ROS directly or by improving the defense system of plants [12]. In the present study, the growth of ginseng hairy roots was promoted at low or moderate levels of Ce^3+^ dosage, but at a high dosage of Ce^3+^, there was a significant promotion of ROS and ginsenoside accumulation. However, a high level of ROS is harmful because the enzymes become less effective for ROS removal and the dynamic balance of ROS in ginseng cells is broken. This conception is consistent with the result of the Ce^3+^-induced suspension cells of *Ginkgo biloba* [46]. It is hence considered that an appropriate level of Ce^3+^ is needed to regulate the accumulation of ROS and secondary metabolism for the healthy and productive growth of ginseng hairy roots.

To protect cells from the damage of excessive ROS, plants have developed an effective ROS-scavenging system. In the defense mechanisms, SOD, CAT and POD play a major role [46]. We studied the gene expression level and enzyme activity of CAT, SOD and POD in ginseng hairy roots (Figure 3D–I). Upon Ce^3+^ treatment for 7 d in a ½ MS medium after a preculture period of 21 d, there was an obvious increase (*p* < 0.05) of SOD activity in ginseng hairy roots when the Ce^3+^ concentration was 10 to 30 mg L^−1^ (Figure 3D). As for CAT and POD activity, they were significant in the Ce^3+^ concentration range of 20 to 40 mg L^−1^ Ce^3+^ (*p* < 0.05) (Figure 3E,F). As expected, the expression levels of the three genes were also induced by Ce^3+^ in a concentration-dependent manner (Figure 3G–I).

Increased activity of antioxidant enzymes, such as CAT, SOD and POD, has been related to protection from oxidative stress in *Pisum sativum* and *Oryza sativa* [47,48,49]. Ce^3+^-treated ginseng hairy roots exhibited a high level of SOD, POD and CAT activities, which could act as circumstantial evidence for its ability to suppress the production of ROS such as O_2_^•^^−^ and H_2_O_2_. Among antioxidant enzymes, SOD catalyzes the dismutation of O_2_^•^^−^ to H_2_O_2_ while CAT and POD catalyze the conversion of H_2_O_2_ to H_2_O [27]. In the present investigation, Ce^3+^ in concentrations of 10–30 and 20–40 mg L^−1^ enhanced the activities of SOD, CAT and POD in ginseng hairy roots, indicating that at moderate concentrations (20 mg L^−1^), Ce^3+^ has advantageous effects on cell growth. At such Ce^3+^ levels, ginseng hairy roots may change their metabolism from growth to defense through higher antioxidant enzyme activities. The increase in SOD activity means that there is a need in ginseng hairy root cells to convert excessive O_2_^•^^−^ into H_2_O_2_ upon Ce^3+^ treatment. The consequent increase in CAT and POD activity might be related to the removal of H_2_O_2_, whose existence is attributable to Ce^3+^ introduction. Similar mechanisms were suggested in the toxicity studies of Cd or Cr on rice [50] and cotton [51]. Overall, the results of the present study indicate that there was activation of protective enzymes in ginseng hairy roots upon ROS production because of Ce^3+^ stimulation. Maintaining a basal level of ROS by using antioxidant enzyme-mediated dynamic equilibrium, which is above a cytostatic level but below a cytotoxic level, therefore enables proper physiological reactions and the regulation of numerous processes essential for life [19]. We speculate that the ROS generated at a moderate dosage of Ce^3+^ were partially degraded by antioxidant enzymes and there was an initiation of secondary metabolism.

### 2.5. Ce^3+^-Induced MeJA Accumulation and Its Biosynthesis Key Enzyme Genes Expression

**Figure 4:**
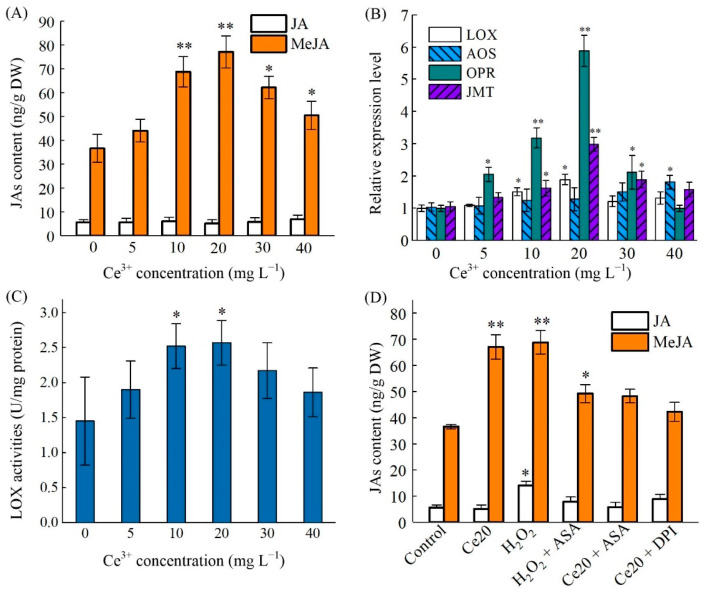
JAs’ accumulation and biosynthesis key enzyme genes expression in ginseng hairy roots. (**A**) JA and MeJA content under different Ce^3+^ treatments for 48 h in a ½ MS medium after a preculture period of 21 d. (**B**) Expression levels of key enzyme genes of JAs’ biosynthesis under different Ce^3+^ treatments for 48 h in a ½ MS medium after a preculture period of 21 d. (**C**) LOX activities in hairy roots under different Ce^3+^ treatments in ginseng hairy roots for 48 h in a ½ MS medium after a preculture period of 21 d. (**D**) JA and MeJA content under different treatments in a ½ MS medium after a preculture period of 21 d. Control, Ce20 and H_2_O_2_ represent treatment by H_2_O, 20 mg L^−1^ Ce^3+^ and 20 mM H_2_O_2_ for 48 h. H_2_O_2_ + ASA, Ce20 + ASA and Ce20 + DPI represent treatment by 20 mM H_2_O_2_, 20 mg L^−1^ Ce^3+^ for 48 h and 1 mM ASA or 10 μM DPI added to the culture 15 min before treatment, respectively. The differences between the treated hairy roots and control hairy roots (0 h) are statistically significant (* *p* < 0.05, ** *p* < 0.01).

Jasmonates (JAs) are phytohormones that have essential functions in plants. They are not only involved in the regulation of plant growth and development, but also participate in the response to environmental changes and external stresses [52]. JAs such as JA and MeJA could be highly sensitive to environmental factors. As shown in Figure 4A, there was no significant change in JA content in ginseng hairy roots upon Ce^3+^ treatments. However, the content of MeJA significantly increased after Ce^3+^ exposures, reaching a peak at the Ce^3+^ concentration of 20 mg L^−1^, corresponding to a 2.1-fold increase in comparison to that of the control (*p* < 0.05).

To determine whether Ce^3+^ can affect the expression of genes related to the metabolic pathway of α-linolenic acid, which could eventually lead to MeJA biosynthesis, we investigated the expression levels of the related genes, including *PgLOX*, *PgAOS*, *PgOPR* and *PgJMT*. The results indicated that these putative genes were activated and upregulated at different levels of Ce^3+^ concentration (Figure 4B). Interestingly, the expression levels of *PgOPR* and *PgJMT* in the MeJA biosynthesis pathway were the highest after Ce^3+^ treatment at a concentration of 20 mg L^−1^, which was highly consistent with the content of endogenous MeJA. The lipoxygenase (LOX) activity of hairy roots was also stimulated by Ce^3+^ treatment, which followed that of MeJA accumulation and reached a maximum value 2.6-fold greater than that of the control (Figure 4C). LOX, one of the key enzymes in JAs’ synthesis, plays an important role in JAs’ accumulation. It was reported that the *PgLOX6* gene from ginseng that encodes a lipoxygenase is responsible for the biosynthesis of JAs and promotion of ginsenosides production through up-regulating the expression of ginsenoside biosynthetic genes [53]. The results showed that LOX activity was activated upon Ce^3+^ treatment, and there was a significant correlation between LOX activity and MeJA biosynthesis. Studies also confirmed that the rapid accumulation of MeJA in plant cells is related to LOX after wounding [54] and fungal induction [55]. Other results also demonstrated that *TaOPR2* was involved in the biosynthesis of JA in wheat [56] and that the overexpression of the *JMT* gene promoted the endogenous MeJA levels in *S. miltiorrhiza* [57].

The results mentioned above supported the hypothesis that Ce^3+^ promotes the accumulation of MeJA by inducing the expression of genes related to JAs’ biosynthesis. The incursion of external metal ions usually induces oxidative stress to plants, rather than directly stimulating the biosynthesis of secondary metabolites and JAs. To investigate whether there was involvement of Ce^3+^-induced ROS in JAs’ accumulation in ginseng hairy roots, we deployed scavengers or inhibitors in our studies. The ginseng hairy roots were pretreated with 10 μM diphenyleneiodonium (DPI, an inhibitor for O_2_^•^^−^ production) or 1 mM ascorbic acid (ASA, a ROS scavenger) before Ce^3+^ exposure. H_2_O_2_ (20 mM), which is an exogenous ROS, was used as a positive control. As shown in Figure 4D, compared with the control (no treatment), the level of JA in ginseng hairy roots did not change significantly after various treatments except for that of H_2_O_2_. The level of MeJA was induced significantly after the addition of Ce^3+^ or H_2_O_2_ and obviously suppressed by the H_2_O_2_ scavenger, indicating H_2_O_2_ directly induced the accumulation of MeJA. The production of O_2_^•^^−^ induced by Ce^3+^ resulted in an increase in the MeJA level, and such a phenomenon was effectively inhibited by DPI because the reduction of O_2_^•^^−^ limited the production of H_2_O_2_. These results suggest that NADPH oxidase is responsible for the Ce^3+^-induced production of ROS, and MeJA biosynthesis is dependent on the oxidative burst. In other words, ROS signifies the activation of MeJA accumulation. This is like the mechanism of low-energy ultrasound-induced JA accumulation in *Taxus* cells [58]. These results have further verified the close relationship of ROS production and JAs’ biosynthesis involvement in mediating the elicitation of ginsenoside production in ginseng hairy roots by cerium. The dependence of Ce^3+^-induced ginsenoside production on JA and ROS production was identified by selective blocking with the corresponding inhibitor and scavenger.

### 2.6. Ce^3+^-Induced PgSS, PgSS, PgDDS Expression and Ginsenosides Biosynthesis

**Figure 5:**
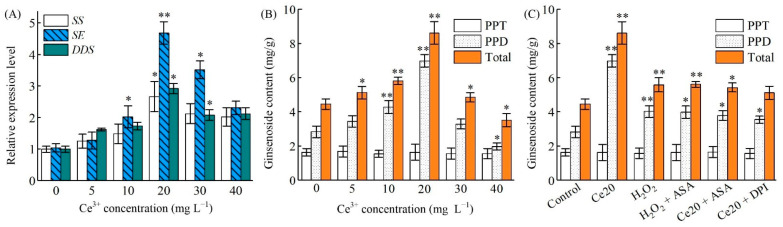
Ginsenosides’ accumulation and gene expression of ginseng hairy roots. (**A**) PPD-, PPT-type and total ginsenoside content under different Ce^3+^ treatments for 48 h in a ½ MS medium after a preculture period of 21 d. (**B**) Expression level of key enzyme genes of ginsenoside biosynthesis under different Ce^3+^ treatments for 48 h in a ½ MS medium after a preculture period of 21 d. (**C**) PPD-, PPT-type and total ginsenoside content in hairy roots under different treatments in ginseng hairy roots for 48 h in a ½ MS medium after a preculture period of 21 d. Control, Ce20 and H_2_O_2_ represent treatment by H_2_O, 20 mg L^−1^ Ce^3+^ and 20 mM H_2_O_2_ for 48 h, respectively. H_2_O_2_ + ASA, Ce20 + ASA and Ce20 + DPI represent treatment by 20 mM H_2_O_2,_ 20 mg L^−1^ Ce^3+^ for 48 h and the addition of 1 mM ASA or 10 μM DPI to the culture 15 min before treatment, respectively. The differences between the treated hairy roots and control hairy roots (0 h) are statistically significant (* *p* < 0.05, ** *p* < 0.01).

Ginsenosides belong to triterpene saponins of which biosynthesis is highly regulated by key rate-limiting enzymes, such as *PgSS*, *PgSE* and *PgDDS* [59]. To further reveal how Ce^3+^-induced ROS promote ginsenoside biosynthesis, the expression level of *PgSS*, *PgSE* and *PgDDS* genes was investigated. As shown in Figure 5A, when ginseng hairy roots were exposed to Ce^3+^ at a concentration of 5–40 mg L^−1^, the expression levels of these three genes changed in a concentration-dependent manner. The expression level of *PgSS*, *PgSE* and *PgDDS* reached maximum at a Ce^3+^ concentration of 20.0 mg L^−1^ (*p* < 0.05), and were 2.66-, 4.68- and 2.92-fold higher than that of the control, respectively. It is worth pointing out that the expression level of *PgSE* was still significant at a Ce^3+^ concentration of 30.0 mg L^−1^. At a Ce^3+^ concentration of 40.0 mg L^−1^, however, there was significant inhabitation of all the gene expressions.

Accordingly, the content of PPT-type ginsenosides (Re and Rg_1_) did not increase significantly after Ce^3+^ treatment (Figure 5B). After 7 d, the content was 9.08 mg g^−1^, only 1.34-fold greater than the control (Table 2). However, the content of PPD-type ginsenosides (Rb_1_, Rb_2_, Rc and Rd) was significantly increased after Ce^3+^ treatment of 48 h, which was 1.77-, 3.01-, 3.52- and 2.05-fold higher than that of no treatment, respectively. After 48 h of treatment, the total ginsenoside increased by 1.71-fold, mainly because the change of PPT-type ginsenosides’ content was small.

**Figure 6:**
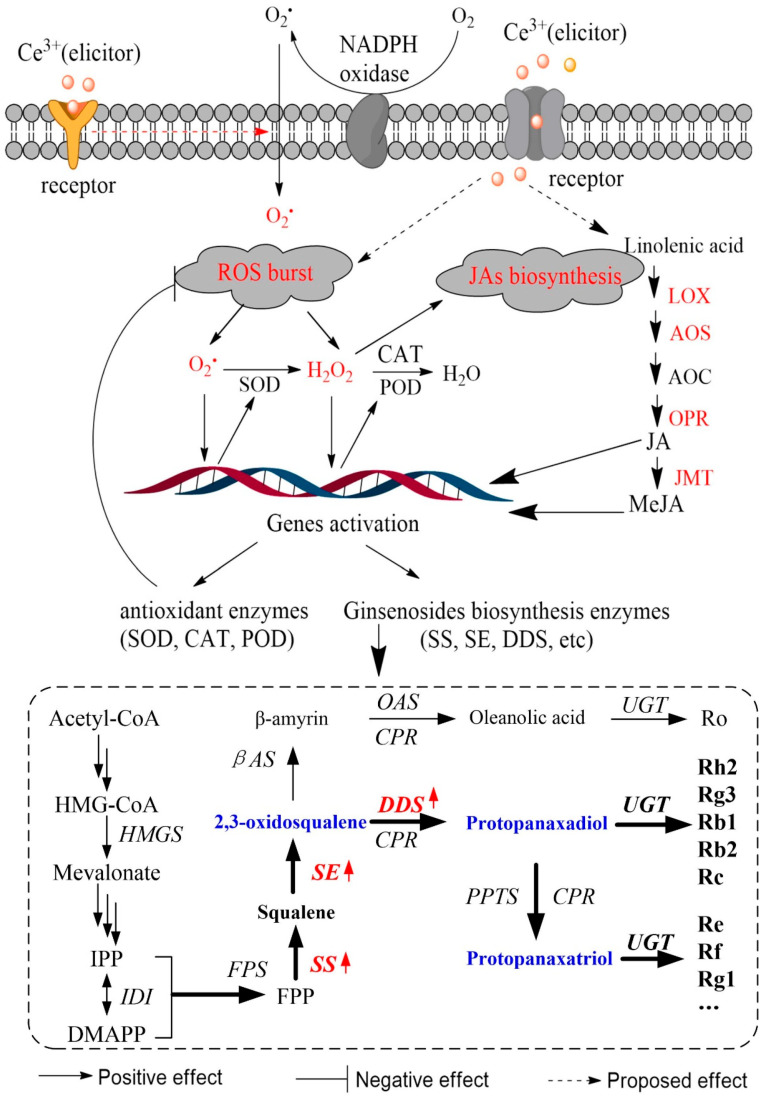
Proposed model for the regulation of ginsenoside biosynthesis following ROS-mediated JAs’ signal pathways. (LOX, lipoxygenase; AOS, allene oxide synthase; AOC, allene oxide cyclase; OPR, OPDA reductase 12-oxophyto-dienoic acid reductase; JMT, jasmonate O-methyltransferase; HMGR, 3-hydroxy-3-methylglutaryl-CoA reductase; FPS, farnesyl diphosphate synthase; SS, squalene synthase; SE, squalene epoxidase; βAS, β-amyrin synthase; OAS, oleanolic acid synthase; CPR, cytochrome P450 reductase; DDS, dammarenediol-II synthase; PPDS, protopanaxadiol synthase; and PPTS, protopanaxatriol synthase; UGT, UDP-glycosyltransferase).

In the present study, the results demonstrated that *PgSS* and *PgSE* were more sensitive than *PgDDS* to Ce^3+^ treatment. This indicates that Ce^3+^ is inclined to regulate the expression of upstream *PgSS* and *PgSE* genes in ginsenosides biosynthesis. Nonetheless, it is impossible to exclude the participation of other important players in this process. JA and its derivatives are known signaling molecules that can induce the biosynthesis of enzymes that are involved in the formation of secondary metabolites in ginseng [60]. Stresses and JAs have been reported to increase the transcript level of *PgSS*, *PgSE* and *PgDDS* [61,62]. Based on the results mentioned so far, one can conclude that the accumulation of Ce^3+^-mediated ginsenosides is induced by the endogenous MeJA-activated upregulation of transcription of *PgSS*, *PgSE* and *PgDDS*. In addition, the expression of these three genes was also induced by incubation of the hairy roots with H_2_O_2_. The inhibitors or scavengers themselves did not affect the transcription of *PgSS*, *PgSE* and *PgDDS* (data not shown). The removal of H_2_O_2_ by ASA or the removal of O_2_^•^^−^ by DPI would result in the inhabitation of Ce^3+^-mediated ROS in the transcription of *PgSS*, *PgSE* and *PgDDS* (Figure 5C). The results disclose that the accumulation of JA-mediated ginsenosides in Ce^3+^-treated ginseng hairy roots is not the only regulation pathway. The proposed model for the regulation of ginsenoside biosynthesis [63] following the ROS-mediated JA signal pathway is illustrated in Figure 6.

It is well known that JA is an important upstream signal for the production of secondary plant metabolites, particularly in the biosynthesis of ginsenosides [60,64]. Environmental stress elevates the level of JA and activates the biosynthesis of nicotine and related pyridine alkaloids in tobacco by up-regulating the expression of genes that catalyzed nicotine formation [65]. The gene expression of key enzymes such as *PgSS*, *PgSE* and *PgDDS* in ginsenoside biosynthesis is related to the content of to-be-catalyzed ginsenosides and is time dependent. Generally, the higher the gene expression level is, the more accumulation of the catalyzed product in the synthesis [66]. The up-regulation of *PgSS*, *PgSE* and *PgDDS* coincided with the biosynthesis of endogenous JA in vanadate-treated ginseng [67]. Our results further imply that the accumulation of Ce^3+^-induced ginsenosides through ROS-induced JA (especially MeJA) biosynthesis is one of the signal transduction pathways for the regulation of ginsenosides biosynthesis. Whether there are other signaling pathways involved in the regulation of ginsenoside biosynthesis still needs to be further studied.

## 3. Materials and Methods

### 3.1. Chemicals, Materials and Treatment

The ginsenoside standards of Rb_1_, Rb_2_, Rc, Rd, Re and Rg_1_ purchased from Chengdu Herbpurify (Chengdu, China) were of chromatographic grade. All the other reagents were analytical grade. Four-year fresh ginseng (*P. ginseng* C.A. Meyer) was collected from Fusong County, Jinlin Province, China. Hairy roots were induced by *A. rhizogenes* A4 [68]. The ginseng hairy roots for cerium treatment were cultured in flasks containing ½ MS liquid medium with an initial inoculation of 1 g hairy roots at 25 °C with shaking at 110 rpm. After a preculture of 21 d, the hairy roots were treated with cerium of different concentrations for 7 d. A ROS scavenger (ASA) [69], an inhibitor (DPI) of membrane NADPH oxidase and the key enzyme for O_2_^•^^−^ production in the oxidative burst [70] were added to the culture 15 min before Ce treatment. After the treatment, the hairy roots were harvested and frozen in liquid nitrogen for RNA, enzymes, JAs and ginsenosides extraction separately.

### 3.2. Determination of Hairy Roots Growth Parameters

After the preculture and Ce^3+^ treatment, the morphology and characteristics of ginseng hairy roots including color and length were monitored. The FW and DW of each sample were measured. The GR (in percentage) of each culture was obtained by dividing the difference between the final FW and initial FW by the initial FW. The DMC of each culture was calculated by dividing the final FW by the final DW [71]. The total yield of ginsenosides was calculated by multiplying the total ginsenoside content by the final FW. The hairy roots were separated by filtration and then dried at 60 °C under vacuum to a constant weight to obtain the dry weight.

### 3.3. Determination of Ginsenosides Content

Ginseng hairy roots were harvested and washed three times with purified water. It was dried to a constant weight at 60 °C for 48 h. Then, the samples were ground to powder. Ginsenosides were extracted using 80% methanol at 60 °C for 1 h in an ultrasonic bath. After filtration, the extracts were washed with ether, followed by extraction with n-butanol. The butanol layer was evaporated to dryness and dissolved in methanol for analysis [68]. The total ginsenoside was filtered by a 0.22 µm membrane filter and analyzed by SHIMADZU LCMS-8050 at 203 nm with a ZORBAX SB-C18 column (3.5 μm, 2.1 mm × 150 mm). The mobile phase consisted of acetonitrile (A) and water (B), and the elute program was as follows: A:B (20:80) for 5 min; A:B (20:80) to (38:62) for 5–40 min; A:B (38:62) to (99:1) for 40–42 min; A:B (99:1) for 42–45 min; A:B (21:79) for 46–56 min [72]. The flow rate was 0.5 mL/min. Rb_1_, Rb_2_, Rc, Rd, Re and Rg_1_ were used as standards. Total ginsenoside was the sum of the ginsenoside components. All the HPLC analyses were performed in triplicate.

The samples were analyzed using an Agilent 6420 triple quadrupole mass spectrometer equipped with an electrospray ionization source. LC-MS analyses were performed in the negative ion mode by a full scan. High-purity nitrogen was used as drying gas (11 L/min) and nebulizer gas (15 psi) spray voltage (4000 V). The atomizing temperature was 300 °C [73].

### 3.4. Determination of O_2_^•^^−^, H_2_O_2_ and MDA

The content of O_2_^•^^−^, H_2_O_2_ and MDA was determined as described before [49]. The content of O_2_^•^^−^ was measured by monitoring the nitrite formation from hydroxylamine in the presence of O_2_^•^^−^. The results were shown based on the change of absorbance at 480 nm min^−1^ g^−1^ fresh weight. The content of H_2_O_2_ was measured using the Ti(SO_4_)_2_ method at 410 nm. The MDA content was assayed by the thiobarbituric acid (TBA) reaction at 532 nm and 600 nm at 25 °C.

### 3.5. Extraction and Assay of Enzyme

The total protein was extracted from ginseng hairy roots as described earlier [74] with slight modification. The fresh ginseng hairy roots were harvested and ground at room temperature after 7 d treatment with Ce^3+^, and then suspended in extraction buffer containing 50 mM Tris-HCl (pH7.4), 150 mM NaCl, 0.1% (*v/v*) Nonidet P-40 and 1 mM PMSF. The homogenate was centrifuged at 4 °C, 5000 rpm for 10 min and the supernatant was collected for determination of the protein concentration using the Enhanced BCA Protein Assay Kit (Beyotime). The activities of SOD, CAT and POD were assayed using commercial kits purchased from the Nanjing Jiancheng Bioengineering Institute (Nanjing, China) [75]. Briefly, the SOD activity was determined by measuring the inhibiting rate of the enzyme to H_2_O_2_ produced by the xanthine morpholine with xanthine oxidase at 550 nm. The CAT activity was measured by the decrease in absorbance at 405 nm due to the decomposition of H_2_O_2._ The POD activity was measured based on the change of absorbance at 420 nm during the catalytic action of H_2_O_2_. Enzyme activities were detected using a UV–vis spectrophotometer (TU-1901, Persee, Beijing, China) at 25 °C. LOX activity was determined by measuring the formation of 13(S)-hydro-peroxylinolenic acid (HPLA) at 25 °C, using linolenic acid as the substrate [58,76].

### 3.6. Measurement of JAs Level

JAs’ (JA and MeJA) levels were determined by Convinced-test Technology Co., Ltd. (Nanjing, China) using HPLC-MS/MS with slight modification [77]. Approximately 1 g of fresh ginseng hairy roots was ground to a powder in a pre-cooled mortar. JAs was extracted using 10 mL of isopropanol at 4 °C for 60 min. After filtration and with an equal volume of dichloromethane, the extract was shaken for 30 min and centrifuged at 12,000 rpm for 5 min at 4 °C. The lower layer was evaporated to dryness under N_2_ and dissolved in methanol (0.1% formic acid) for analysis. The sample was filtered by a 0.22 µm membrane filter and analyzed by HPLC-MS/MS. HPLC analysis was performed using an Agilent ZORBAX SB-C_18_ column (3.5 μm, 2.1 mm × 150 mm), eluted with solvent A consisting of methanol−0.1% formic acid and solvent B consisting of ultrapure water/0.1% formic acid as the mobile phase at 45:55 (*v/v*). MS conditions were as follows: The spray voltage was 4500 V; the pressure of aux gas, nebulizer and air curtain was 70, 65 and 15 psi, respectively; and the atomizing temperature was 400 °C.

### 3.7. Genes Expression Analysis of Antioxidant Enzymes, Key Enzymes of JAs and Ginsenosides Biosynthesis

The expression levels of antioxidant enzyme genes including *PgSOD*, *PgCAT* and *PgPOD*, key enzyme genes of JAs’ biosynthesis including *PgLOX*, *PgAOS*, *PgOPR* and *PgJMT* and key enzyme genes of ginsenoside biosynthesis including *PgSS*, *PgSE* and *PgDDS* were quantified by qRT-PCR using the SYBR Green stains on the Mini Opticon real-time system (Bio-Rad, Hercules, CA, USA). Total RNA was extracted from ginseng hairy roots using Plant RNA Kit (Omega, Doraville, GA, USA). Reverse transcription was performed using HiFiScript gDNA Removal RT MasterMix (CoWin Biotech Co. Ltd., Beijing, China). The relative expression level was shown after normalization with *β-actin* and calculated using the formula 2^−ΔΔCt^ [74]. All qRT-PCR reactions were performed in triplicate. The primers are listed in Table 3.

### 3.8. Statistical Analysis

The results were presented as mean ± standard deviation (SD) values of three replicates. All the data were statistically analyzed by one-way analysis of variance (ANOVA) with SPSS (version 17.0, Chicago, IL, USA), followed by the Duncan test. A *p*-value of less than 0.05 was regarded as significant.

## 4. Conclusions

The culture of hairy roots is a promising alternative to improve the production of target secondary metabolites through various elicitors or precursors supplemented in the media. The present study provides the first evidence that the optimized use of Ce^3+^ on a ½ MS medium can act as an effective elicitor to enhance the biosynthesis of pharmaceutically active ginsenosides in hairy root cultures of *P. ginseng.* The main mechanism is that Ce^3+^ activates the biosynthesis of ROS-mediated endogenous MeJA, which induces the accumulation of ginsenosides. Meanwhile, the presence of Ce^3+^ in a concentration of 10 mg L^−1^ in the culture medium resulted in high growth of hairy roots and biomass accumulation, and a concentration of 20 mg L^−1^ resulted in a high yield of ginsenosides. For in vitro production of medicinal herbs with efficacy, the growth parameters and biomass accumulation should be coupled with the efficient biosynthesis of active constituents. It is demonstrated that the proper use of Ce^3+^ can lead to improvements in both growth indices and the yield of valuable active compounds in the culture of *P. ginseng* hairy roots.

## Figures and Tables

**Table 1:** Effect of Ce^3+^ concentration on the growth of ginseng hairy roots.

Ce Concentration mg L^−1^	Hairy Roots Growth Parameters				
	Average Root Length (mm)	Fresh Weight (g)	Dry Weight (g)	Dry Matter Content (%)	Growth Ratio
0	3.6 ± 0.1 ^a^	9.2 ± 0.2 ^a^	0.8 ± 0.2 ^a^	8.2 ± 0.3 ^a^	8.0 ± 0.5 ^a^
5	4.0 ± 0.2 ^b^	12.7 ± 0.3 ^b^	1.1 ± 0.1 ^b^	8.4 ± 0.2 ^a^	11.2 ± 0.7 ^b^
10	5.1 ± 0.2 ^c^	16.0 ± 0.5 ^c^	1.7 ± 0.1 ^c^	10.8 ± 0.8 ^b^	13.9 ± 0.7 ^c^
20	5.0 ± 0.1 ^c^	14.8 ± 0.6 ^d^	1.6 ± 0.2 ^c^	11.1 ± 0.9 ^b^	13.3 ± 0.8 ^c^
30	3.6 ± 0.2 ^a^	10.7 ± 0.4 ^e^	1.0 ± 0.2 ^b^	9.6 ± 0.5 ^c^	9.2 ± 0.6 ^d^
40	3.4 ± 0.3 ^a^	8.1 ± 0.3 ^f^	0.7 ± 0.1 ^a^	8.5 ± 0.3 ^a^	6.7 ± 0.5 ^e^

Data are average values of three replicates ± standard deviation (SD). Means in each column with the same letters are not significantly (*p* < 0.05) different based on Duncan’s Multiple Range Test.

**Table 2:** Ginsenoside content in ginseng hairy roots under Ce^3+^ treatment for 7 d in a ½ MS medium after preculture of 21 d.

Ce^3+^ (mg L^−1^)	Ginsenoside (mg/g FW)						Total Content(mg/g)	Total Yield (mg)
	Rb_1_	Rb_2_	Rc	Rd	Rg_1_	Re		
0	2.2 ± 0.08 ^a^	0.3 ± 0.01 ^a^	0.4 ± 0.06 ^a^	1.9 ± 0.1 ^a^	2.0 ± 0.1 ^a^	2.9 ± 0.1 ^a^	9.6 ± 0.13 ^a^	88.7 ± 2.1 ^a^
5	2.5 ± 0.07 ^a^	0.4 ± 0.02 ^b^	0.6 ± 0.08 ^b^	2.2 ± 0.1 ^a^	2.1 ± 0.1 ^a^	3.1 ± 0.3 ^a^	10.9 ± 0.4 ^a^	137.8 ± 5.2 ^b^
10	3.6 ± 0.2 ^b^	0.5 ± 0.06 ^b^	1.3 ± 0.1 ^c^	3.0 ± 0.3 ^b^	2.6 ± 0.2 ^b^	3.2 ± 0.4 ^b^	14.2 ± 0.5 ^b^	227.6 ± 8.3 ^c^
20	3.9 ± 0.3 ^b^	0.9 ± 0.08 ^c^	1.4 ± 0.1 ^c^	3.9 ± 0.2 ^c^	2.8 ± 0.1 ^b^	3.6 ± 0.3 ^b^	16.4 ± 0.5 ^c^	242.2 ± 5.6 ^d^
30	3.8 ± 0.2 ^b^	0.8 ± 0.04 ^c^	1.4 ± 0.1 ^c^	3.6 ± 0.2 ^b^	2.8 ± 0.3 ^b^	3.4 ± 0.4 ^b^	15.8 ± 0.3 ^c^	168.8 ± 6.4 ^e^
40	2.0 ± 0.1 ^a^	0.3 ± 0.05 ^a^	0.3 ± 0.06 ^a^	1.9 ± 0.1 ^a^	2.0 ± 0.1 ^a^	2.9 ± 0.3 ^a^	10.1 ± 0.2 ^a^	81.7 ± 4.2 ^a^

The data are the average value of three replicates ± standard deviation (SD). Means in each column with the same letters are not significantly (*p* < 0.05) different based on Duncan’s Multiple Range Test.

**Table 3:** Primers of the selected genes verified by qRT-PCR analysis.

Gene	Primer	Sequence (5′ → 3′)
*PgSOD*	Forward	CTAAACCCCTCACCGTCGTC
	Reverse	TTCACTGTAGTTGGGCCGTC
*PgCAT*	Forward	AGATACCGGACTTTTGCGCC
	Reverse	GACACCCATATGCTGCGGAT
*PgPOD*	Forward	GGATTCTTCCACCGCTCCAA
	Reverse	CCTTTGTCGGCGAACGTTTT
*PgLOX*	Forward	CGTGGTGGACAGAAATCCGA
	Reverse	TTGAGGGGTTTTGAGGACGG
*PgAOS*	Forward	CTGCAGGTGGAATTAGCGGA
	Reverse	CATCCGGAGCGATTCGTACA
*PgOPR*	Forward	GATGGCTCTAGCGTTGCAGA
	Reverse	TGACTGTTAACACCTACCGGC
*PgJMT*	Forward	ACAGGAGGCCGAATGGTTTT
	Reverse	CCTTAAGGGCGACGGCTAAT
*PgSS*	Forward	TAGGCATGCGGAAAAGCAGA
	Reverse	TGTTGAATGACGAGGCCGAA
*PgSE*	Forward	TCGCGATCTTCTTAGGCCAC
	Reverse	CCACTGGCTTGCGTAATGTG
*PgDDS*	Forward	CCCTGCAGTGCCTACTGTTA
	Reverse	CTCCCAAACTGCGAAACCAC
*RolB*	Forward	GCTCTTGCAGTGCTAGATTT
	Reverse	GAAGGTGCAAGCTACCTCTC
*RolC*	Forward	CTCCTGACATCAAACTCGTC
	Reverse	TGCTTCGAGTTATGGGTACA
*β-actin*	Forward	TGCCCCAGAAGAGCACCCTGT
	Reverse	AGCATACAGGGAAAGATCGGCTTGA

## Sample Availability

Samples of the compounds are available from the corresponding authors.
